# Disruption, Slowness, and Collective Effervescence: Children’s Perspectives on COVID-19 Lockdowns

**DOI:** 10.1007/s42448-022-00147-4

**Published:** 2023-01-16

**Authors:** Tobia Fattore, Gabrielle Drake, Jan Falloon, Jan Mason, Lise Mogensen

**Affiliations:** 1 School of Social Sciences, Macquarie University, Sydney, Australia; 2 School of Social Sciences, Western Sydney University, Sydney, Australia; 3 School of Medicine, Western Sydney University, Sydney, Australia

**Keywords:** Children’s experiences of COVID-19, Childhood vulnerability, Children and time use, Children as citizens

## Abstract

The COVID-19 pandemic represented not only a health crisis, but a social crisis for children, one that has disrupted notions of what a good childhood is. However, the longer-term implications of the pandemic are still to be seen, for children, their families and communities. This article is concerned with what these ongoing changes may be, based on a qualitative multi-stage study that asks children about their experiences of well-being before the pandemic, during lockdowns and post-COVID-19 lockdowns. This included asking seven children in online semi-structured interviews about what aspects of life brought on by COVID-19 restrictions they would like to see continue post-lockdown. We outline some of our findings. We describe new rituals and ways of organising time developed by children, facilitated by the use of digital technologies. We describe these new ways of managing time as task-based rather than rule-based, with children experiencing slowness of and greater control over their time. We found that lockdowns provided a possibility for children to assert a public agency through banal acts of sociability, for example, by conforming to public health measures such as mask-wearing and hand-washing. Whilst small acts, children discussed these in terms of being moral agents (protecting the safety of others) and as part of a larger civic attitude they observed around them. Thus, their acts can be seen as expressions of larger forms of social solidarity that contributed to a sense of collective effervescence.

## Introduction

The COVID-19 pandemic represented not only a health crisis, but a social crisis for children, one that has disrupted notions of what a good childhood is. UNESCO (2022) declared that the disruptions caused by the pandemic, especially to children’s education, have created a lost generation, a group perpetually disadvantaged because of the lost years of the pandemic. However, the longer-term implications of COVID-19 on generational outcomes are yet to be seen, and a kind of orthodoxy of ‘living with COVID’ has begun to emerge, with pre-pandemic ways of life returning.

This article is prompted by concerns about the enduring effects of the pandemic on children and is based on early findings from a study where we are tracking children’s perspectives of their well-being prior to, throughout pandemic-related lockdowns and post-lockdowns, in Sydney, Australia. Specifically, we asked our participants a somewhat provocative question, that being ‘what aspects of life that changed during the pandemic would they like to see continue?’ This question, as opposed to many other questions asked of children about their experiences of COVID-19, emphasises not only the importance of top-down measures and negative impacts of lockdowns, but how children responded and continue to respond to the pandemic, and attempts to capture the complex ways in which individuals, households and communities responded to the COVID-19 crisis.

This article documents some of the responses that emerged from our study. Within the intimate and private sphere, we find children developing new rituals and ways of organising their time, facilitated by the use of digital technologies. Whilst initially the pandemic was experienced as a shock to daily habits, for our participants, lockdowns eventually were experienced as involving a slower pace where time use was associated with tasks rather than rules. Consequently, children were able to assert more control over how they used their time. This contrasted with the lack of control that children, or any private citizen, could exert over time in the public sphere, with the race against the spread of the disease occurring in frenetic and dramatic ways.

We found that lockdowns and associated regulations imposed by the Australian government provided possibilities for children to assert public agency through banal acts of sociability. These included the micro-rituals of personal hygiene, social distancing and mask-wearing, public health behaviours advocated by governments as critical to stopping the spread of the disease. For our participants, these behaviours represented a commitment to public civicness and an expression of them being moral agents. In part, this commitment reflected their concern that rather than being vulnerable themselves, they could put others at risk, by being vectors for disease spread. Moreover, by undertaking these behaviours, children self-consciously located themselves within larger social efforts to combat COVID-19, which reflected and contributed to a sense of collective effervescence, in the Durkheimian sense. Whilst referring to moments where members of a society come together to perform a religious ritual to celebrate the ‘sacred’ and demarcate the sacred from the banality of daily life and habits (the profane), Durkheim (1912/1965) pointed out that the essential experience of collective effervescence involves moments where the group comes together and communicates or acts with the same sentiments and behaviours, which serve to unify a group of individuals and celebrate the group itself. The shared emotion that is generated serves to elevate the individual into a state of being that may be euphoric. These moments are replicated not only in religious ceremonies, but civil rituals, and in spontaneous moments of collective celebration or mourning (Bellah, 1967). We argue that such feelings — of the celebration of the group and in an emotional elevation of the individual as part of the collective experience of the pandemic — is evident in children’s reflections on their moral agency during the pandemic.

Therefore, whilst the COVID-19 pandemic intensified children’s vulnerability, we demonstrate that, for some children at least, greater ability to control time in the private sphere and engage in everyday acts of solidarity, which were more outward facing, were ways our participants tried to deal with the significant disruption and anxiety produced by the pandemic. This article proceeds by reviewing some of the literature that documents children’s vulnerability during the pandemic, before outlining our research and presenting the findings from our study.

## Constructing Terrains of Vulnerability: Children and COVID-19

The COVID-19 pandemic has re-focused attention on the multi-dimensionality of children’s vulnerability and marginality. The effects have been broad ranging and have impacted deeply. Rates of poverty, unemployment, mental health problems, substance abuse, child abuse and neglect and intimate partner violence have all been reported to have risen during the pandemic (Finch & Hernández Finch 2020, Green, 2020, Hyde, 2020, Kutsar & Kurvet-Käosaar, 2021, United Nations Office and Drugs and Crime, 2020). As noted, significant educational disruption occurred during the pandemic (Sibieta, 2021). Among the measures used to implement social distancing and reduce the spread of the virus, large numbers of schools were closed. UNESCO estimated that over 190 countries had closed their schools nationwide, and several other countries implemented regional or local closures, affecting the education of 90% of children worldwide (see UNESCO, 2022). UNESCO warned that these closures are likely to widen the learning gap between children from lower-income and higher-income families, with children from low-income households being far less likely to have the resources to make home-based learning viable — resources like a reliable internet connection, access to a computer device or a suitable place to do homework.

The economic impacts have also been significant. Save the Children and UNICEF (2021) estimated that 150 million additional children were pushed into poverty due to the impact of the pandemic. We know that the impacts of poverty are different for children compared to adults. In its most severe form, poverty leading to malnutrition can have long-term consequences on children’s physical, social and emotional development. Immediate loss of income means families are less able to afford the basics required to sustain children’s development, including access health care and education. These effects are exacerbated where fiscal contraction results in the withdrawal of services which many families depend on. For the poorest families, the lack of access to social care services or income support measures limited their ability to conform to containment and physical distancing measures and thus further increased their exposure to infection.

Data is also emerging about the impacts on children’s mental health (Dubois-Comptois et al., 2021; Elharake et al., 2022, Newlove-Delgado et al., 2021). The COVID-19 pandemic may worsen existing mental health problems and lead to more cases of poor mental health among children and adolescents. Increased anxiety about the future, isolation from peers, exposure to cultures of fear and calamity may be factors that contribute to poor mental health. In particular, changes in routines caused by school closures, social distancing and home confinement due to COVID-19 potentially disrupt predictability and security in everyday life. One study involving 3613 Chinese students from 20 provinces found that levels of anxiety in children and adolescents during the epidemic were much higher than before the pandemic, with the prevalence of clinical depression symptoms being 9% higher than general estimates (Duan et al., 2020). In Italy, a study by Spinelli and colleagues (2020) emphasises contextual factors, finding that quarantine’s impact on children’s behavioural and emotional health is mediated by parent’s stress levels. In Australia, self-reports indicate that psychological well-being, including ability to cope and levels of stress and anxiety, was negatively affected during the pandemic. Similarly in England, an increase in mental health problems since the pandemic began has been reported, with a five-percentage point increase for children aged 5–16 years reporting mental health problems (Newlove-Delgado et al., 2021).

These findings direct our attention to children’s vulnerability and specifically their vulnerability to educational disruption, poverty and its consequences and adverse mental health outcomes (see also Stoecklin et al., 2021). This vulnerability is not equally shared, with those with the least resources most likely to be impacted by the broad ranging effects of COVID-19. This emphasis on the increased vulnerability of children during the COVID-19 pandemic raises a different spectre, as vulnerability can be used to justify unwarranted paternalism and coercion of individuals and groups identified as vulnerable. In Australia, both the national and state governments put in place significant measures to control the spread of the virus. These included strict domestic and international border closures; extensive quarantine measures for returning travellers, people who contracted the virus and their close contacts; an extensive contact tracing system; and periods of total lockdown (with the lockdown of Melbourne, the capital city of Victoria, being one of the world’s longest, lasting 111 days). Australia was one of the few countries in the world that, prior to the development of the vaccine, effectively and continuously implemented a suppression strategy. At the height of the virus prior to the roll-out of vaccinations, in June 2021, the 7-day average of new cases across the nation was 12 new cases only. Australia ranked 123rd in the number of cases and 105th in the number of deaths globally.

The policy response framed children in particular ways. As outlined by Fattore et al. (2021), these included an *institutional* framing that emphasises the role of educational institutions in organising children’s experiences of the pandemic, evident in school closures and the shift to home-based schooling; a *developmental frame* that raises concerns about the effects of COVID-19 on children’s development, apparent in concerns about children’s absence from school, leaving them vulnerable to exploitation or engaging in criminal activity (UNODC, 2020), and a *pathological frame*, where time at home in isolation, away from peers and the routines of school life, focused attention on children’s mental health and their capacity, or more aptly lack of capacity, to be resilient. Yet, in terms of health vulnerability, the rate at which children obtain the infection remains lower than adults, and children are more frequently asymptomatic or experience only mild symptoms (CDC, 2021). This, and the focus on certain forms of vulnerability in the framing of children and COVID-19, attests that an understanding of the implications of the lockdown for children’s ‘lifeworlds’ from their perspectives is only beginning to emerge.

## Methodology: Researching Living the Pandemic with Children

This study was undertaken as part of the *Children’s Understandings of* *Wellbeing: Global and Local Contexts* (CUWB) study (see Fattore et al., 2019), a qualitative, multinational study which seeks to examine how children conceptualise and experience well-being from a comparative and global perspective. The study involves 31 teams from 25 countries that uses a shared research protocol and a set of overarching principles. These principles include that:

- Children are seen as social and moral actors who can provide narratives about their experiences of well-being and of everyday life.
- Children’s perspectives must be understood within the social and cultural orders which they are part of. This also means being sensitive to values and norms and explicitly analysing how values and norms are part of enacting cultural contexts.
- Researchers are co-constructors of meaning along with participants, requiring that the research process and the social relations between children and researchers needs to be reflected upon carefully (Fattore et al., 2019).

The Australian team adapted the CUWB protocol for a sub-study of children’s experiences of the pandemic, utilising child-centred and participatory techniques, including task-oriented methods, such as drawing, photography, making a digital movie and mapping. This was part of a sub-study of a larger study on children’s well-being involving 106 children aged between 8 and 16 years. Children for the major study were recruited from five different locations with differing socio-demographic characteristics, including areas with high levels of urban and rural populations, diversity of socioeconomic status and distinct ethnic groups.

The COVID-19 sub-study reported on in this article includes two elements: (1) fieldwork with children and (2) fieldwork with significant adults. As we only report on the interviews with children in this article, we will only discuss this part of the study.

Seven children and young people aged 10–17 years participated in in-depth, online interviews (see Table 1). Children were recruited via purposive and snowball sampling techniques (Merriam & Tisdell, 2015), through personal and professional contacts, such as peak child advocacy organisations, sporting clubs and youth services. Consent to participate was obtained from both the child and a parent/carer, and the research only proceeded if consent was obtained from both. Children could withdraw from the study at any time without any reason needing to be given, and the researcher actively monitored for signs of distress or boredom that might indicate that the child was potentially uncomfortable with continuing their participation in the research. This situation did not arise in this part of the fieldwork.

**Table 1:** Characteristics of children who participated in the study

*Name*	*Age*	*Gender*	*Ethnicity*	*Socioeconomic status*	*Location*
Ruth	10 years	Female	Australian	Low	Metro Sydney
Harriet	12 years	Female	Australian	High	Metro Sydney
Nick	14 years	Male	Vietnamese	Low	Metro Sydney
Paul	12 years	Male	Maori	Medium	Metro Sydney
Alex	17 years	Male	Australian	Low	Metro Sydney
Violet	15 years	Female	Australian	Medium	Metro Sydney
Riya	10 years	Female	Indian	High	Regional NSW

*NSW, New South Wales eastern Australian state

Qualitative fieldwork with children was undertaken over two phases.

### Phase 1: Before, During and After Social Isolation

Phase 1 obtained thick descriptions of children and young people’s daily lives before social isolation measures were in place, during social isolation and after social isolation measures loosened. Via an in-depth, online interview, participants were asked to describe their daily experiences during these periods. Additionally, they were asked what they were looking forward to and would miss as social isolation measures loosened and what their fears and hopes for the future were as related to the end of lockdown and other public health measures.

### Phase 2: Documenting Changes in Everyday Life

At the end of phase 1, participants were asked if they would like to take part in a second stage where they would discuss their experiences of the loosening and end of public health measures, in particular those related to stay-at-home orders. More specifically, they were asked to record any experiences they deemed significant and different in regard to a range of topics (important things, people, places, family, friends, dimensions of change) compared to their experience when full lockdown measures were in place. For this stage, participants were given the option to create a short film/video documenting their insights and/or experiences; create a set of ‘digital focus’ stories showcasing their experiences of change; or keep a video, photo or written diary of experiences of change. After a period of 4–8 weeks, a phase 2 interview occurred. These were open interviews which involved the participant discussing what their creation is and interpreting this for the researcher. Therefore, the interviews allowed the participant to prioritise topics they thought were important or significant, with the researcher.

Interviews lasted between 45 and 90 min with breaks as required. Prior to the interview, consent was reconfirmed, and consent to record the interviews was discussed. In all cases, the participant agreed for the interview to be audio-recorded. Interviews were digitally recorded, transcribed and thematically analysed using Braun and Clarke’s (2012) methods of data familiarisation; the generation of initial codes; identification, review and revision of themes; definition and naming themes; and lastly, producing the final analysis.

## Findings

### Disruption and Slowness: Children’s Experiences of Time During Lockdowns

The pandemic disrupted the usual routines and habits of everyday life, with the closure of workplaces and schools requiring the majority of the population to remain at home. This involved a dissolution of boundaries between work and home life and, for children, between school and home life, where the majority of children were required to switch to home-based schooling. Consequently, lockdowns meant that children, their teachers and parents had to reorganise their daily routines around schooling quickly and in major ways. Whilst daily routines are usually taken for granted, their disruption made them visible and subject to question. Time itself became an issue, specifically whether pre-pandemic ways of managing time were the best way of organising daily life (see also Levrini et al., 2021).

Initial fears were that the closure of schools and workplaces might lead to a further intensification and acceleration of the experience of time, with parents having to juggle care and work responsibilities, whilst children had to take greater management of their learning. These concerns echoed theorists such as Giddens (1990), Beck (2000) and Lash (2002) who argue that the pace of life and of change itself has accelerated, resulting in a displacement of fixed social bonds by bonds of communication, such as those mediated by digital technologies. Such concerns are further elaborated by Hartmut Rosa (2013) who described modern society as a ‘society of acceleration’ (Rosa, 2013). Rosa argues that acceleration in the pace of life has an effect on identity and subjectivity, with a sense of time as having direction, being replaced by a sense of time being directionless, that whilst we are constantly engaged in activities, we nonetheless feel inert, and regardless of the effort, we do not experience a sense of progress. Parenting is a clear example of this, with the expectation to manage and curricularise children’s time, both during school hours and outside school hours, becoming an evaluative norm of parenthood (Lareau, 2011). From this perspective, we see how an acceleration in the pace of life feeds concern about the mental health of children, with the hurried child evoking images of the stressed and anxious child.

The acceleration of time was evident in the spread of the disease itself and attempts to manage it by national governments and supra-national bodies such as the WHO. These organisations were in a race with the disease — a race to close borders and find a vaccine pitted against the spread of the disease, counted in the number of new cases and breakout locations. This race was materialised by the metronome of daily press briefings by health ministers and epidemiologists, which provided a regular time marker of daily life through the pandemic. The frantic pace of the public battle against the virus was paralleled for some in the intensification of life brought about by the need to juggle work and family life, especially for parents of school-aged children, who were now also obliged to manage their children’s education. Yet for others, particularly those who lost their jobs, time stalled. For these people, the pandemic transformed time-based routines in ways that disrupted and potentially reversed time acceleration as described by Rosa and others.

For the children in our study, the pandemic created an initial sense of disruption, with anxiety being expressed about what the restrictions would mean for living, as Ruth^1^ expresses, a ‘normal life’:

I feel like it’s a little different because there’s still all those restrictions and it’s kind of like you get used to having a normal life and then you go into isolation, and you used to have—and going into isolation and then we finally get out. And also, it’s weird because it just keeps changing and all that. (Ruth, female, 10 years)

Interviewer: What do you think it was that overwhelmed you at first because you were saying that you thought, it is actually the same amount of work, but you felt overwhelmed?

Harriet: There was so much tasks and I was like can I get this done in time? What happens if I don’t and thinking lots of negatives. So, then I kind of stopped thinking the negatives and tried going on the positives, and so that really helped me do my work. (Harriet, female, 12 years)

Both participants note the disruption of going into and out of lockdowns and the uncertainty created by readjustments during the initial lockdown stages. Harriet expresses a commonly held sentiment of anxiety, caused by the uncertainty of not being sure whether she could time manage all the tasks required (‘can I get this done in time’), despite being the same amount of work.

However, our participants expressed a new relationship with time once the initial shock of lockdown passed, establishing new routines. They contrasted their experience of the hectic nature of daily life prior to the pandemic, with time being much slower during the lockdowns. There was a sense from our participants of *returned time*, of more time being available for self-care, reflection and self-directed learning. The heterotopic aspect of this is evident in the following quotes where children compared daily routines before and after lockdowns commenced. We include two extracts from an interview with Paul (12 years), where he outlines a typical school day before lockdowns with one during lockdown, and an extract from Nick (14 years) and Harriet (12 years) who also discuss the slowness of COVID-19 related lockdowns.

It was just sort of a normal day. I’d get up. I’d have the rush in the morning. Get all your stuff ready. Find that you didn’t have your sport uniform packed or something. And then quickly get out the door ready for the bus. And then I’d get to school, and then catch the bus home. And I’d just do some homework when I got home. So, it was quite frantic, and it was very busy and go-go. So, I found isolation definitely slowed the things down, and it was very peaceful in the mornings especially, being able to roll out of bed and start school.

…

So, a typical day would generally look like, I’d probably wake up pretty early because that was how I used to wake up to go to school. I’d wake up and I’d say good morning to my family, and I would probably just go on a walk with my mum, and we’d go for probably half an hour walk before school. And then I’d normally go back home and sit at my desk for a little while and do some work. I would either get up and get myself some breakfast, or I would start a Zoom. And then for the first part of isolation we didn’t have Zooms, so it was a very flexible day. I could just wake up early, get it all done. If I was feeling tired I could sleep in and do it later. So, after school I would just chill out with my family. Maybe play some games and just really relax with the family. I really enjoyed social isolation. I felt I was able to spend more time with my family, which is really nice to have everyone at home. (Paul, male, 12 years)

Being able to be at, like, home. Sometimes it’s fine with school, like, you’re rushing around, like a lot, getting on buses and, like, getting to class on time, and I found with online learning you were just able to have a bit of like a slower paced life. You could get up later and start school later but also like, just click a link to get to your next class which I found really helpful. (Nick, male, 14 years)

Waking up late, sleeping in and not having to do much in the mornings, because in a normal morning before Coronavirus I’d have to brush my teeth, go to the toilet, sometimes comb my hair, clean my room, make sure it’s neat and tidy and make sure you smell nice, but now I just have to brush my teeth, breakfast. So yeah, that was one of the best parts, and another one would be I could eat my breakfast during school which was also a cool thing. (Harriet, female, 12 years)

These descriptions are testaments to new rituals that were developed because there was more time available to them, time was returned. The language used by Paul belies the pace of life before and during the pandemic. The rapid fire ‘And then … And then…’ is replaced by a longer and more languid description of life. The compulsion of needing to do all that is required to get to school (rush, pack, bus, school, home, homework) is replaced by descriptions of choice over tasks made possible by having more time. Nick explicitly contrasts ‘rushing around’ with a ‘slower paced life’ and Harriet describes how having more time allowed her to do more tasks involved with self-care.

Participants also discussed how lockdown provided them with more time from structured commitments, so that returned time could be used for leisure activities, as outlined by Alex (a phenomenon not specific to children, as the amount of documented sourdough baking and ukulele playing taken up by adults during the pandemic attests to):

We got to spend a lot of time hanging out with friends online on a PlayStation or on Zoom. It was that time, with family as well, that we don’t normally get during a school year. You could walk out to the kitchen in the middle of the day and have a good conversation with your parents. (Alex, male, 17 years)

However, what emerged as more significant for our participants time use than unstructured activities was how school activities were used by our participants to organise daily activities, much in the same way working from home organised time for parents. Violet, 15 years, provides an illustrative example of the methodical ordering of time and the development of new routines around the school day which occurred during lockdowns. However, like Harriet, Nick and Paul, there is no rush. Rather, the ordering of time allows time for self-care, reflection and perhaps developing different or new aspects of one’s subjectivity. In this respect, time ordering is only loosely correlated with chronological time and is more associated with functional time that is the time required to do tasks rather than time determined by externally imposed systems, such as the formal school or work day:

Yeah. So, I really tried to wake up earlier, like around 6.00 maybe, when I woke up early, I had a better structured routine. Some days I would wake up at 7.00 or so but we kind of start school around 8.20. So, I liked – I did like having that initial two hours to really prepare myself for the day. So, I woke up around 6.00, I would try and get up, kind of do my little morning routine, my skincare, breakfast, I then dressed myself. Then after that I would start Zoom. I would typically Zoom for about seven hours a day.

So, we had our first two periods of Zoom and then we have tutor group, which was a time when girls from - like two or three girls from every year kind of come together and they’re known as a group, so that we either have that or we have recess straightaway. So, during recess I would quickly have a snack and then just go for like a half an hour or 15-minute kind of walk, just to give me like a bit of exercise. Then I would Zoom for another two and a half hours or so and then after that I would have lunch. During lunch it was kind – I kind of found I really just wanted to relax and have a mental break from Zoom, get some food and then after that I went for another walk with my parents or with my family, just trying to break it up, unless they had meetings of course. After that I then had another two hours of Zoom. Then any little bit of work we didn’t complete during the Zoom class we had to complete at the end of the day, and at the end of the day it was just time for homework and kind of just trying to revise everything that I’ve learnt and just understand the work more and don’t forget about it. (Violet, female, 15 years)

Home-based schooling then for many of these children provided some control over time, what Levrini et al. (2021) describe as a private re-appropriation of daily time, bringing aspects of life within control or at least having a sense of agency over how to spend one’s day. This re-appropriation is especially critical given the lack of control individuals in general could exert over the ‘world outside’, whether because of the regulations associated with the lockdown or the spread of disease which necessitated these restrictions. Moreover, this control over time complicates the image of educational disadvantage usually associated with COVID-19-related lockdowns.

### Blurring the Public and Private: Children’s Use of Digital Platforms During Lockdowns

In contrast to early sociological analyses of digital technologies that see such technologies as grafted onto existing social relations, more recent analyses of digital technologies have argued that it is impossible to separate the social from the technological and that digital technologies, like all technological innovations that have preceded them, are significantly constituted by and adapt to existing social relations (Third et al., 2019). For our participants, the use of digital technologies became integral to social interactions during lockdowns, being the way that relationships with extended family and friends could be maintained where physical contact was no longer possible.

As the quotes we have included so far demonstrate, home-based schooling was only made possible through the use of digital technologies. However, their use did not necessarily involve a smooth transition, with the use of technologies like Zoom, having to be learnt and adapted to by our participants. As Violet points out in the following quote, online digital platforms were only useful where they facilitated control over time, meaning she could learn according to her preferred style and pace, for Violet being able to communicate directly with teachers and getting on with tasks — what some educationalists would refer to as Violet’s ‘learning style’ (Pashler et al., 2009). However, Violet also expresses frustration where the technology is used to enforce pedagogical styles that undermine her ability to control her own learning. When Violet refers to ‘everything in breakout’, she is expressing a sentiment shared by many adults and children, of experiencing online meeting fatigue (Döring et al., 2022).

I found Zoom a very great program to use, just because I found that the program allowed me to interact with my teachers, but I found it a very distracting program as well just because I could be getting on with my work and then students would come online and ask a question and it kind of just made me not necessarily focus on my work. I definitely saw a difference in like my motivation to study and the motivation to do work after Zoom. Even though it was two days with the lesson plan, it was kind of enough to set me in that routine straightaway and then it was just this sudden change of Zoom and then I would Zoom for seven hours a day. It was just – it’s not – where I felt that the lesson plans were so much better for me personally, but I know Zoom did help others, but I found the lesson plans just – I can understand the work. I could just email my teacher if I had any questions, or we could set up another Zoom. I just found it difficult – I did like Zoom when they were explaining the assessment task but during isolation and online learning, I found it really difficult to complete assessments, just because the group work was on Zoom. Everything in breakout. (Violet, female, 15 years)

Digital platforms were also used by children to maintain social relationships — extending the intimate sphere virtually — and maintain engagement in community activities. In this sense, the use of these technologies made the distinction between public and private more porous. In the following interview extracts, Violet discusses the use of online platforms to keep in touch with extended family whilst Ruth discusses the importance of these technologies to stay in contact with friends and continue one of her extra-curricular activities, her dance lessons.

Yeah. So, we – during like restrictions–to stay connected we Zoom a lot for like major celebrations or just kind of times where we felt a bit disconnected. Yeah, I definitely missed my grandma, my grandparents and my aunties and uncles and cousins. Yeah, it was kind of just a time where I interacted differently with them, if that makes sense. I didn’t feel disconnected from them, but I felt like due to COVID everyone again, was in the exact same boat. It wasn’t just a few people; we were all there. So, in that kind of sense I didn’t feel majorly disconnected from my family but of course I did miss them. (Violet, female, 15 years)

Interviewer: Okay. And how was it different in terms of your friends? Because obviously, you know you’re not on the playground.

Ruth: Yeah, definitely not. Probably just like video calling and stuff like that. Yeah waving through Messenger, and saying hi and everything on chat. … It was different and also harder ’cause I—you never knew—you would never know when they were available. You know like at school you could say, “Hey, are you available right now? Do you want to talk?” And then like in isolation, you could just basically send a message and see if they respond.

Interviewer: And you were saying before that, you know, you do a lot of dancing and so you’re very active during the week. What did that look like during online learning? Did you have to stop that altogether or did you do it by Zoom?

Ruth: We did it by Zoom. But it was very, very difficult because—so like I had to dance in a small living room rather than in a studio. We have a massive studio that we can dance in and also because that—we didn’t really get to learn that much on Zoom like we learned like the middle of a dance and then we got up to about a quarter left and usually we’d be finished by that time. Yeah all of my dance Eisteddfod’s, we only had one and I was only doing two dances out of six. (Ruth, female, 10 years)

Our participants raise both the possibilities and limits of these technologies for them during lockdowns. Digital technologies allowed children to keep in contact with extended family, friends and maintain their activities, in ways similar to face to face contact but with some additional capacities. By compressing time–space, children were able to see friends in different geographical locations simultaneously. By being an instantaneous technology, children could reach out throughout the day and make connections with friends who were also online. By using the screen as an interface, physical spaces became multi-functional, with the living room becoming a dance studio. Yet there are evident limits in these technologies as well. The embodied and multi-sensory elements of being with someone were missing — as Violet states ‘it was kind of just a time where I interacted differently with them’, and as Ruth points out, video calls do not substitute for being together on the playground. Moreover, there are limits to which a small living room can substitute for a dance studio, and this impacts on both the experience of the activities and what is possible to be achieved. As Ruth points out when discussing her dance lessons, ‘we didn’t really get to learn that much on Zoom’.

Wajcman (2008) points out the ability of technologies, such as email and online meeting and learning platforms, to invert the relationship between time and space, meaning that activities such as school lessons or dance class do not necessarily have to be primarily designated to physical places, such as the school or dance hall, but rather are designated with specific allocations of time, when people meet virtually to undertake the activity together. Many concerns have been raised regarding the implications of this time–space inversion, from the desolation of urban centres to the dissolution of feelings of community, resulting from what Wajcman describes as the potential for these technologies to ‘foster a “mobile privatization” or “networked individualism” that is not tied to workplaces or home time–space boundaries’. (Wajcman, 2008 p. 68). For children, this ‘mobile privatisation’ has been expressed in concerns about their increasing exposure to online harm (Moody et al., 2021). However, we found that the functionality of digital technologies allowed children to maintain connections with friends and family, albeit in a less than desired way. It appears that the institutionally mandated use of digital platforms for educational purposes, enforced by school closures, laid some groundwork for greater acceptance of children’s use of digital platforms for other purposes, such as being with friends. Whilst the literature on home-based schooling has largely focused on the pedagogical effectiveness of these technologies or how home schooling deepens inequalities in educational experience, very little has focused on how home schooling represents the extension of children’s public sphere responsibilities into the private sphere of their homes (the exceptions including Barn & Sandhu, 2022 and Fegter & Kost, 2022, this issue), something which, from our findings, is central to children’s experience of the pandemic.

### Being a Responsible Citizen: Micro-rituals of Care During the Lockdown

Whilst much of the pandemic has focused on the mental health crisis, individuals and communities also demonstrated greater levels of sociability in response to the pandemic, with acts of understanding and kindness towards strangers, expressions of solidarity with front-line workers, especially healthcare workers, and the use of humour to make fun of ‘living with COVID’, being documented as ways of coping with the pandemic around the globe. The 2022 World Happiness Report recorded an increase in volunteering, donating to charity and helping a stranger during 2021 compared to before the pandemic (Helliwell et al., 2022). These behaviours demonstrate what O’Brien (2021) describes as ‘a manifest democratic sense of togetherness, … despite the belief in the association between pandemics and scapegoating, violence and blame, they are in most cases linked with a new commitment to interdependence and a lack of blame for an ‘other’’ (p. 113).

Given this context of goodwill, it is perhaps unsurprising that our participants discussed the importance of acting according to public health measures as a personal and civil duty. In the following extract, Violet describes a cognitive understanding of the seriousness of the pandemic (‘thousands of people were dying a day and that was kind of a big recogniser for me to understand that, hey COVID is really a real thing’), which is for her an important rationale for complying with public health measures. Whilst her own personal safety is important, in discussing her compliance with public health measures, she describes the devastating social and economic impacts of the pandemic:

Before COVID started and it was declared a pandemic I didn’t take it as seriously as I did coming in – like in isolation I knew it was serious because we had to go into isolation, but the after effect of COVID it kind of really like that hundreds of thousands of people were dying a day and that was kind of a big recogniser for me to understand that, hey COVID is really a real thing. … So, yeah, like most – particularly up the road it’s pretty much just this row of local businesses and you could just tell that there was no atmosphere, it was just closed and especially on cold winter days, it kind of just really felt like, it just felt dark and even though people were walking, especially going down this road of local businesses, it felt kind of sad. It just felt like this is someone’s business, this is someone’s life, and a global pandemic has just – they’re all open now, and they’re all thriving, but it kind of just made me realise that local businesses are definitely going to struggle, and I feel like cafes, and like flower shops and news agencies and stuff like that, it’s kind of really made me appreciate this is someone’s livelihood, you know. (Violet, female, 15 years)

Whilst Australia enforced some of the harshest lockdowns in the world, public support for these measures was quite high (Faasse & Newby, 2020; Tranter, 2022). Similarly, our participants agreed with the measures taken by state and federal governments. Whilst Harriet and Ruth both point out the strictness of the rules, Harriet describes them as ‘a bit over the top’ and Ruth ‘does not like them at all’; nonetheless, they advocate for the measures as a way to control the spread of the disease. Harriet describes then as being important so that ‘Australia doesn’t get Coronavirus country’ and Ruth describes them as ‘good cause it kept us safe’:

Interviewer: What do you think about the government’s rules about social isolation?

Harriet: Well I find them pretty fair and square even if they go a bit over the top it’s like they’re doing them for our own good so that we don’t get it, so that Australia doesn’t get Coronavirus country.

Interviewer: And is there anything that you’re hopeful about in terms of thinking about things that might have been changed. Is there anything that’s made you hopeful, things that we can take out of our time during lockdown or our time during the Coronavirus that we want to commit to or inform how we live our lives in the future?

Harriet: Probably hopeful for the amount of effort given to making the vaccine. I see people are trying to put their whole life into the vaccine, so that’s one thing I’m really hopeful and thankful about actually. (Harriet, female, 12 years)

Interviewer: Okay. And what did you think about these rules? So, what did you think about why the government was making us do this? What did you think about the reasons why they were making us stay at home, or do online learning?

Ruth: I think it was actually really smart of the government because it was a lot of COVID cases that were coming in during that time. And I just think it was a good idea, but then again, I actually didn’t like them at all, because you couldn’t do anything normal. So, I think it was good ‘cause it kept us safe, but it was bad because we weren’t living a normal life. (Ruth, female, 10 years)

Zinn (2021) describes these kinds of attitudes and the public support for lockdown measures as ‘herd humanism’ (p. 445), which he contrasts with the policy strategy of herd immunity, that translate rational calculative methods for risk management into everyday risk practices. Trust is a critical component for this process of translation, which combines a cognitive (knowledge) and affective (relationship) dimension (Barbalet, 2009; Zinn, 2021), and we see this emerge in children’s discussions of the public health orders and what was required of them — based on trusting governments to manage the spread of the disease, and for Harriet trust in the scientific community to develop a vaccine for the disease.

One of the more obvious expressions of this ‘risk work’ was in everyday routines around hygienic practices, which participants discussed as a way that they can contribute to reducing the spread of disease, as an act of social solidarity to support public health measures. Whilst much literature has emphasised children’s *exposure to risks of the pandemic*, and therefore as vulnerable, our participants also emphasised another element — of them *managing the* *risks of the pandemic*. Harriet and Ruth both describe their ongoing commitment to hygiene and sanitising to maintain public health, despite the inconvenience:

Whenever we enter the classroom we always wear hand sanitiser and we leave with hand sanitiser, whenever we do anything we always wear hand sanitiser. And it got a bit frustrating, but then again, we were like, okay, this is for our own good to stop germs from spreading. (Harriet, female, 12 years)

Interviewer: Are you still doing things that we did when we were in lockdown before?

Ruth: Oh yeah, definitely. Like there’s like wash your hands about like ten times a day, sanitise your hands quite a few times and its just kind of annoying. Like you keep doing it over and over and over again when like before you didn’t have to sanitize your hands as much or wash your hands as much. (Ruth, female, 10 years)

Whilst social distancing, mask-wearing and hand sanitising involve the juridical and scientific disciplining of bodies (Durnová & Mohammadi, 2020, Pfaller, 2020), they are also examples of everyday etiquette, which link state imperatives with private responsibility in a way that expresses and affirms our self-understanding of being a private citizen belonging to a public collective. And because of children’s constrained agency, everyday rituals of sociability cannot be underestimated in their importance. These have been described by O’Brien (2021) as micro-level rituals, everyday acts of sociability which also include things like playing music to neighbours, decorating front gardens and other ways to raise the morale of others, which provide ‘a muted, festive quality to the lockdown’ (O’Brien, 2021, p. 114).

### Public Fear and Celebration: Being a Vector of Disease Amidst Collective Effervescence

Another dimension of this commitment is that whilst children harboured fears for their own health from contracting COVID-19, they more frequently expressed concern about spreading the virus to others, and that they, as children, represented a potential health risk to other more vulnerable people. As O’Brien notes, the abstract notion of numbers of cases and deaths has a highly embodied and private nature to it, in that ‘Our nostrils, earholes, mouths – the doors from the monad to the social – have become sites of danger, requiring protection, distance and cleansing to prevent the infectious outside world penetrating it’ (O’Brien, 2021, p. 116). Our participants were concerned that ‘their nostrils, earholes and mouths’ had become sites of danger to others. This worry, that *they are the risk*, was an important reason why our participants emphasised how important it was for them to comply with public health orders. This concern is clearly expressed by Alex and Nick. Alex discusses his risk aversion, not wanting to infect others because of his participation in social activities, whilst Nick explains that his cautious behaviours (‘remember not to touch the escalator or, you know, definitely don’t put your hands near your mouth’) are important to keep his elderly grandparents safe:

Interviewer: Are there things that you worry about with COVID sticking around? Are there things that you worry about for your own health or family’s health or your life and opportunities in the next little while?

Alex: Yeah. I definitely worry for everyone’s health, especially in our family, just hopeful. Most people are vaccinated—everyone’s vaccinated so that’s a relief. You don’t want to put other people at risk because of your wants or social life. I don’t want to have to go out on a weekend and then have to come back and worry about putting people in my family at risk. That becomes a big thought in the back of your mind while you’re out and then it’s those sort of health things. (Alex, male, 17 years)

I think with my family, we’ve definitely been taking a lot more precautions and we’re alot more aware of germs and, you know, you know like, being very cautious about, you know, like we have elderly grandparents. So, you know, being careful if we're going to go see them, wearing a mask and then also like being vaccinated. Um, I definitely say I’m a lot more like worried and aware of germs because for so long you’re trying to remember not to touch the escalator or, you know, definitely don’t put your hands near your mouth. … . I’m not in a dangerous situation, I don’t have any underlying health issues, but just because of COVID and the impact that it can have on people’s lives. I’m being a lot more cautious and a lot more aware of the germs you can catch. (Nick, male, 14 years)

These anxieties can be understood as an emotional reaction to a concern that they, by inadvertently breaching or violating public heath rules, restrictions and orders, might endanger the safety of others. The converse of this commitment is then anger and frustration towards those who flagrantly break the rules which are meant to safeguard others. However, children’s altered behaviours around hygiene and care and their awareness that they are the risk point, intensified their sociability. Things like, as we have noted, playing music to neighbours, decorating front gardens and celebrating front-line workers, which were observed during lockdowns were expressions of Durkheimian collective effervescence (Durkheim, 1893/1964, 1912/1965) or Honneth’s (2007) notion of solidarity of mutual concern. Whilst not overly religious, these acts involved moments where people came together, whether on their balconies, backyards or online, to engage in collective rituals expressing shared sociability.

This was commented upon by children in our research. Whilst the demands to engage in, for example, education and public health behaviours as we have described earlier are somewhat stylised, this was compensated with the joy and relief that was associated with increased informal sociability and care evident in their neighbourhoods during the lockdowns and after lockdowns ended. Nick describes the outpouring of friendliness that resulted from the shared experience of lockdowns and how this collective behaviour ‘help[ed] stop the spread’. Paul describes looking forward to the future, where ‘people will become a lot more friendly’, because of these shared experiences:

Interviewer: And how about people in the community. What do you think has changed for people in the community, compared to before COVID and now?

Nick: I’ve definitely noticed there’s a lot more friendliness, like we’ve all been through this together, and you know we’re all a community and we’re all part of this. We all have to help stop the spread, like we all have to have a part in stopping this and helping each other and you know, looking after everyone and I definitely think like, you know, it’s just been like the community. (Nick, male, 14 years)

I feel just anyone that you pass in the street now you’ll just know – something to relate to, we’ve both been through the COVID pandemic. And I’m very hopeful that things will settle down. And I’m just – I feel I’m looking forward to the future because I feel people will become a lot more friendly because of, as I was saying, people were smiling and talking, so I feel everyone’s just had to find different ways to talk to people. And I remember around my neighbourhood they did this thing where they put bears up in their houses, and kids would go on treasure hunts. And I hope we can do more little things that help the community and help parents with kids and stuff like that. (Paul, male, 12 years)

These sentiments are similar to the kinds of cathartic emotional responses to other collective events, like the end of war, which complicates the depiction of children as victims of the pandemic. In their concern for others, expressed practically through complying with health and hygiene orders and emotionally in that they are concerned about infecting others, children are enacting agency around living with COVID-19. They are agents who are responsible for their and others health, involved in reciprocal care relationships. Therefore, whilst these behaviours reflect some anxiety about how the pandemic might develop in the future and whether what they can do will be enough, this is tempered by a sense of optimism, or as Paul puts it, ‘people smiling and talking …and I hope we can do more little things that help the community and help parents with kids and stuff like that’.

## Discussion and Conclusion

Living through and with the COVID-19 pandemic has undoubtedly created stressful situations for individuals. As lockdowns were enforced, our existing routines proved to be ‘unreliable’ for the new situation, causing disruption and in many instances anger, anxiety, stress and fatigue. Yet, for our participants, new routines emerged that provided some relief from the pace and hectic routine of life before the pandemic. Our participants developed an altered relationship with time, which not only helped them manage life during lockdowns but in some ways was preferable to pre-COVID routines. However, it is unlikely that this can be understood as a uniform experience. Much more work is required to understand how this altered relationship with time is socially distributed. We know that this is dependent on access to other socially valued resources, like income, appropriate housing and being digitally enabled. Moreover, within households, it is likely that children’s experience of slowness is highly dependent upon their parents, largely mothers’, active management of work and care responsibilities. It is quite likely that for our participants, their new experience of time required their parents to frantically juggle domestic and work responsibilities. Therefore, we must be sensitive to differences in resources available to different children based on their social position and be aware that children’s experiences are reliant on the informal care and unpaid domestic work provided by their carers, which remains highly gendered (Krase et al., 2021).

Our findings indicate that the permeability of public and private spheres, which has been accelerated by the increasing use of digital technologies, intensified for our participants during COVID-19-related lockdowns. The reliance on technology necessitated the acceptance of changed behaviour arising from the division between the public and private in areas such as school and leisure. Moreover, the institutionally mandated use of digital platforms for education laid some groundwork for the greater acceptance of children’s use of digital platforms for other purposes, such as being with friends. Whilst much research on the use of digital technologies has been concerned that they provide a mechanism for colonisation of the private sphere by, for example, the world of work (an ‘on-call culture’) and for children, concerns about exposure to online harms (Third et al., 2019), our participants pointed out that these technologies were neither a saviour nor curse. Whilst they allowed children to continue with many aspects of their life, the experience of education, leisure and relationships online fell somewhat short of the multi-sensory experience of being in the same physical place with other people.

Further, whilst the pandemic experience has been read in terms of social emotions, this reading has not been applied to children, with the emotionality of the pandemic for children being largely concerned with adverse mental health outcomes, some of which have bordered on fatalism. Our participants certainly expressed fears about future outbreaks and about infecting others, but they also emphasised the collective effervescence they observed in their communities and which they were part of, especially when feeling they were acting morally by doing their bit to keep others safe. Just as much as there was shared fear, there was also solidarity, empathy and altruism. Moreover, their concern about being a vector of disease and acting responsibly to avoid putting others at risk inverts how we normally think about childhood vulnerability, in that children were the risk and others were vulnerable because of them. For our participants, their behaviours were not framed in terms of ‘resilience’, but responsibility — they could do things to keep others safe.

## Data Availability

The data generated during and/or analysed during the current study are not publicly available due to the ethical requirements for this study.

## Footnotes

1 All participant names are pseudonyms allocated by the authors.
